# Novel ribotype/sequence type associations and diverse CRISPR-Cas systems in environmental *Clostridioides difficile* strains from northern Iraq

**DOI:** 10.1093/femsle/fnad091

**Published:** 2023-09-18

**Authors:** Srwa J Rashid, Janet Y Nale, Andrew D Millard, Martha R J Clokie

**Affiliations:** Medical Laboratory Technology Department, Koya Technical Institute, Erbil Polytechnic University, Erbil, Kurdistan, Iraq; Centre for Epidemiology and Planetary Health, Scotland’s Rural College, Inverness IV2 5NA, UK; Department of Genetics and Genome Biology, College of Life Sciences, University of Leicester, University Road, Leicester LE1 7RH, UK; Department of Genetics and Genome Biology, College of Life Sciences, University of Leicester, University Road, Leicester LE1 7RH, UK

**Keywords:** *Clostridioides difficile*, multilocus sequence typing, whole-genome sequencing, phylogenetic analysis, CRISPR-Cas system, prophage

## Abstract

The environment is a natural reservoir of *Clostridioides difficile*, and here, we aimed to isolate the pathogen from seven locations in northern Iraq. Four of the sites yielded thirty-one isolates (ten from soils, twenty-one from sediments), which together represent ribotypes (RTs) 001 (five), 010 (five), 011 (two), 035 (two), 091 (eight), and 604 (nine). Twenty-five of the isolates (∼81%) are non-toxigenic, while six (∼19%) encode the toxin A and B genes. The genomes of eleven selected isolates represent six sequence types (STs): ST-3 (two), ST-15 (one), ST-107 (five), ST-137 (one), ST-177 (one), and ST-181 (one). Five novel RT/ST associations: RT011/ST-137, RT035/ST-107, RT091/ST-107, RT604/ST-177, and RT604/ST-181 were identified, and the first three are linked to RTs previously uncharacterized by multilocus sequence typing (MLST). Nine of the genomes belong to Clade 1, and two are closely related to the cryptic C-I clade. Diverse multiple prophages and CRISPR-Cas systems (class 1 subtype I-B1 and class 2 type V CRISPR-Cas systems) with spacers identical to other *C. difficile* phages and plasmids were detected in the genomes. Our data show the broader diversity that exists within environmental *C. difficile* strains from a much less studied location and their potential role in the evolution and emergence of new strains.

## Teaser

*Lactobacillus casei* condition medium downregulates miR-21 relative expression in HT-29 colorectal cancer cell line, which can potentially be related to induction of apoptosis in cancerous cells rather than in healthy cells.

## Introduction

The nosocomial *Clostridioides difficile* infection (CDI) is characterised by antibiotic induced diarrhoea and pseudomembranous colitis (Czepiel et al. 2019). The identification of clinical *C. difficile* ribotypes (RTs) in environmental settings indicates putative connections with humans and animals and could contribute to the emergence of new strains in hospital and thus pose a significant health risk (Janezic et al. 2016, Czepiel et al. 2019, Williamson et al. 2022). Environmental *C. difficile* strains that are genetically related to those isolated from human in clinical settings suggest that the same strains can inhabit multiple niches and the environment is a reservoir of CDI (Knight et al. 2017, Rodriguez Diaz et al. 2018, Janezic et al. 2020, Lim et al. 2020, Williamson et al. 2022). Core genome single nucleotide variant (SNV) analysis revealed that 42% of human strains showed clonal relationship (separated by ≤2 SNVs in their core genome) with one or more strains from environmental samples (Knight et al. 2017, Janezic et al. 2020). This strongly supports a persistent community reservoir with long-range dissemination. Since the sources/reservoirs outside the hospital setting play a significant role in the transmission of CDI, continuing molecular and genomic surveillance of strains from these sources is vital to find opportunities to reduce the overall CDI burden (Knight et al. 2017, Lim et al. 2020).

*Clostridioides difficile* diversity is mainly characterized using PCR ribotyping, which distinguishes the strains based on the size and copy number of the 16S-23S rRNA intergenic spacer region (Indra et al. 2008, Chatterjee and Raval 2019). Polymerase chain reaction (PCR) ribotyping is clearly useful for outbreak investigations (Seth-Smith et al. 2021) and has relatively equal discriminatory power to multilocus sequence typing (MLST), which identifies *C. difficile* strains based on the combinations of seven unique housekeeping genes that allow designation of allele profiles or sequence types (ST) to represent a genotype (Griffiths et al. 2010, Knight et al. 2017, Janezic and Rupnik 2019). Whole genome sequencing (WGS), however, permits single nucleotide-level strain resolution over all genomic space, thus, it is essential for long-term epidemiological, evolution, and population dynamics studies (Dominguez et al. 2016, Dingle et al. 2017, Muñoz et al. 2017, Uelze et al. 2020). WGS is currently accessible due to the low sequencing cost and availability of publicly available genome data, which provide valuable resources for more in-depth genome comparisons than ribotyping.

*Clostridioides difficile* surveillance is more effective in western countries but very few epidemiological studies are reported in northern Iraq, which leaves a significant geographic lack of awareness of this bacterium in this part of the world. We have reported the genomes of three novel *Clostridium* sp. strains isolated from the environment in northern Iraq (Rashid et al. 2016), but, to date, no other environmental *C. difficile* genomes from this region have been reported. This highlights the paucity of knowledge that exists on strains such as RTs 001, 010, 011, and 035 that are circulating in the environment in this part of the globe and their potential role in clinical settings (Hargreaves et al. 2013, Hargreaves et al. 2016, Janezic et al. 2016). To further knowledge in this area and strengthen the existence of clinically relevant *C. difficile* strains in the natural environments, here, we isolated and genetically characterized environmental isolates from northern Iraq. We included strains from our previous studies to conduct whole-genome analyses to ascertain their RT/strain type relationships. Furthermore, we analysed the diverse CRISPR-Cas systems found within the strains and compared these features to strains from other regions to better ascertain possible genetic interactions that occurred through horizontal gene transfer via prophage elements and their role in *C. difficile* evolution.

## Materials and methods

### Sampling sites

To isolate *C. difficile* from northern Iraq, soil (seven) and sediment (five) samples were collected from seven sites: Hamamok, Dokan, Jalee, Chnarok, Taq Taq rivers, and Safeen and Haibat Sultan mountains between 2012 and 2013 (Supplementary Table S1). Samples were collected into screwed-capped, sterile falcon tubes, immediately stored at 4°C, and processed within 2 weeks of collection.

### Recovery of *C. difficile* isolates from environmental samples

*Clostridium difficile* was isolated using previously described enrichment procedures (Hargreaves et al. 2013). Briefly, ∼1 g of soil/sediment was mixed with 10 mL of fastidious anaerobic broth supplemented with 250 µg mL^−l^ cycloserine and 8 µg mL^−1^ cefoxitin (Bioconnections, Leeds, UK) to select for *C. difficile*. Also, 0.1% sodium taurocholate (Sigma–Aldrich, Dorset, UK) was added to the enrichment to enhance spore germination (Foster and Riley 2012). The cultures were incubated for 10 days in a MiniMACS anaerobic chamber (Don Whitley Scientific, West Yorkshire, UK; 10% H_2_, 5% CO_2_, and 85% N_2_) at 37°C, then centrifuged for 10 min at 5000 × *g*. To further select for *C. difficile* spores and reduce other bacterial contaminants, the pellet was treated with an equal volume of industrial methylated spirit and incubated for 30 min at room temperature. A loopful of the mixture was spread on Brazier’s cycloserine, cefoxitin, and egg yolk (CCEY) selective agar plates and incubated anaerobically for 48 hours. *Clostridioides difficile* colonies were purified through three further rounds of sub-culturing on Brain Heart Infusion (BHI) agar (Oxoid, Ltd., UK) supplemented with 7% defibrinated horse blood (TCS Biosciences, Ltd., UK). The presumptive colonies were identified by the characteristic horse manure smell, colony morphology, and yellow-green fluorescence under the long-wave ultraviolet light (Delmée 2001). The isolates were confirmed by PCR targeting the *C. difficile* 16S rRNA gene, as described by Rinttilä et al. (2004). Bacterial isolates were stored in Protect bacterial preservers (Technical Service Consultants, Ltd., Heywood, UK) at −80°C.

### Ribotyping and toxin gene characterization *C. difficile* isolates

To determine if multiple RTs were found in each site or sample, ten randomly selected bacterial isolates from each sample were subjected to conventional and capillary PCR ribotyping targeting the intergenic spacer 16S-23S rRNA genes using primers GTGCGGCTGGATCACCTCCT-3′ and 5′-CCCTGCACCCTTAATAACTTGACC-3′ (Indra et al. 2008). DNA was extracted from broth cultures that were grown for 18–24 hours anaerobically using 5% Chelex® (BioRad Laboratories, California, USA). The PCR ribotyping conditions were denaturation at 95°C for 120 s, followed by 30 cycles of denaturation at 92°C for 60 s, annealing at 55°C for 60 s, elongation at 72°C for 90 s, and a final extension at 72°C for 5 min. PCR products alongside a 100-bp DNA ladder (Fermentas, York, UK) were resolved at 3%. Response regular agarose gel (Geneflow, Staffordshire, UK) prepared in 1 × Tris-acetate—EDTA buffer and stained with GelRed (Biotium, Hayward, California, USA; Nale et al. 2012). Images were visualized using the SynGene application in a UV transilluminator. Fragments from capillary ribotyping were analysed using Peak Scanner software v1.0 (Applied Biosystems, UK). The similarity of the strains was assessed using a MultiVariate Statistical Package (MVSP, Kovach Computing Services, Anglesey, UK) based on the presence of amplicons of a particular size. Sorensen’s distance was calculated between each combination of isolates and clustered (Supplementary Fig. S1 and B; Shan et al. 2012, Nale et al. 2016). Eleven strains from the sites with six distinct patterns of amplicons were submitted to Leeds Reference Laboratory, UK for further confirmation of RT designation and toxin genes presence. All the isolates were further screened for the presence of the toxin genes using multiplex PCR with primer pairs NK2 and NK3, which amplified the partial sequences of *tcd*A, NK9, and NK11 targeting the essential repeat region within the *tcd*A (Kato et al. 1998) and NK-104 and NK-105 for the toxin B gene (Barroso et al. 1990). Amplification conditions for the multiplex PCR for the NK primers were initial denaturation at 95°C for 5 min, followed by 32 cycles of denaturation at 95°C for 20 s, annealing at 62°C for 120 s, elongation at 72°C for 2 min, and a final extension step at 72°C for 5 min. Binary toxin genes *cdt*A and *cdt*B presence were determined using primers and procedures previously described (Barroso et al. 1990, Stubbs et al. 2000). PCR reaction conditions were initial denaturation at 95°C for 5 min, followed by 30 cycles of denaturation at 94°C for 45 s, annealing at 52°C for 60 s, elongation at 72°C for 2 min, and a final extension stage for 5 min at 72°C. PCR amplicons were resolved in a 1% molecular-grade agarose gel (Bioline, UK) in 1xTAE with GelRed and visualized as described above.

### Whole-genome sequencing

To further assess the genome diversity within the isolates, a total of eleven isolates comprising of three isolates of RT091, two isolates each of RT001, RT035, and RT604, and one isolate each of RT010 and RT011 were sequenced using the Illumina MiSeq (2 × 250 bp paired end) platform following NexteraXT library preparation. The genomic DNA was prepared from broth cultures that were grown for 18–24 hours anaerobically in BHI broth (Oxoid, Hampshire, UK) using a QIAGEN Genomic Kit according to manufacturer’s instructions. Approximately 1 ng of DNA was used in the Nextera XT DNA sample preparation (Illumina, San Diego, California, USA), following the manufacturer’s instructions. Libraries were sequenced using a MiSeq V2 reagent kit (2 × 250 bp). Genomes were assembled using SPAdes 2.0 with the following parameters: ‘-k 21, 33,55,77,99127 –careful’. All genomes were submitted to the European Bioinformatic Institute (EBI) and Enterobase under the project accession PRJEB8702. The genome can be accessed online at: https://www.ebi.ac.uk/ena/browser/view/PRJEB8702. Contigs were ordered against the reference strain *C. difficile* CD630 (NC_009089) using MAUVE v2.3.1 (Darling et al. 2004). Genomes were annotated using PROKKA v1.14.5 with the following settings: ‘– compliant – genus *Clostridium* use genus’ (Seemann 2014). *Clostridioides difficile* isolates were sequence-typed as previously described by Griffiths et al. (2010), utilizing seven regions within conserved the housekeeping genes (*adk, atpA, dxr, glyA, recA, sodA*, and *tpi*). Alleles from the assembled genomes were extracted and queried against the curated *C. difficile* database (https://doi.org/10.12688/wellcomeopenres.14826.1; Jolley et al. 2018). To ascertain the phylogenetic relationships between the new isolates and strains from different global locations and relevant additional *C. difficile* strains, we chose the genomes of 78 *C. difficile* strains that are publicly available on Enterobase (http://enterobase.warwick.ac.uk) and NCBI based on their strain types, diverse sources, and geographic locations (Supplementary Table S2). A maximum likelihood tree was constructed using PhyML (Guindon et al. 2010) as described previously (Didelot and Wilson 2015). Recombination was accounted for using ClonalFrameML (Didelot and Wilson 2015). The tree was visualized in iTOL software v6.4.3 (Letunic and Bork 2019).

### Prophage carriage prediction

Predictions of prophage encoded in the genomes of the 11 sequenced strains were determined using PHASTER (PHAge Search Tool Enhanced Release; using default parameters; Zhou et al. 2011, Arndt et al. 2019). Prophages were detected by querying of contigs against viral and prophage databases in Genbank. PHASTER hits were automatically classified into intact (score > 90), questionable (score 70–90), and incomplete (score < 70) prophages based on their sizes, similarity to known phages, and the presence of phage-like and phage cornerstone genes (e.g. ‘capsid’, ‘head’, ‘plate’, ‘tail’, ‘coat’, ‘portal’, and ‘holin’; Greenrod et al. 2022).

### CRISPR arrays prediction

To establish the diversity of the CRISPR-Cas system within the genomes of the isolates, array prediction was conducted using PILERC-CR 1.06 with default settings (Edgar 2007, Ekseth et al. 2013). Direct repeat (DR) sequences were aligned in the Clustal Omega (Sievers et al. 2011) to establish consensus sequences and viewed with Jalview v2 (Waterhouse et al. 2009). The webserver PADLOC was used to determine the CRISPR-Cas system types within the genomes of the isolates based on profile Hidden Markov Models (Payne et al. 2021). Identified spacers were searched against Genbank and NCBI nucleotide BLAST and RefSeq-Plasmid databases to identify a possible extrachromosomal origin using the CRISPRTarget tool (Biswas et al. 2013). The default values used by NCBI BLASTn for short sequences, <30 bases (defaults for long sequences are in brackets) are: gap open −5(−5), gap extend −2(−2), match + 1(+1), mismatch −10(−10), minimum score 30 (Biswas et al. 2013).

## Results

### *Clostridioides difficile* was isolated from four of the seven sampling sites

Of the seven sites sampled, only four (Dokan, Jalee, Hamamok, and Chnarok) yielded *C. difficile*, of which 31 isolates were recovered from these samples (Table 1, Supplementary Table S1, Fig. S1). We did not isolate *C. difficile* from Taq Taq river (one soil and one sediment samples), Safien mountain (two soil samples), and Haibat sultan mountain (one soil sample) despite sampling Safien mountain twice in the summer and winter of 2012 and 2013, respectively.

**Table 1.** RT designation and toxin gene carriage of isolates examined in this study.

RT	Isolates	Site	Sample	Genotype	ST	Clade
604	K3	Hamamok	Soil	A¯B¯CDT¯	ND	ND
	K1	Hamamok	Soil	A¯B¯CDT¯	ND	ND
	K6	Hamamok	Soil	A¯B¯CDT¯	ND	ND
	K7	Hamamok	Soil	A¯B¯CDT¯	ND	ND
	K9	Hamamok	Soil	A¯B¯CDT¯	ND	ND
	K10	Hamamok	Soil	A¯B¯CDT¯	ND	ND
	7F	Hamamok	Soil	A¯B¯CDT¯	ND	ND
	CD105KSO7	Hamamok	Soil	A¯B¯CDT¯	177	C-I
	CD105KSO8	Hamamok	Soil	A¯B¯CDT¯	181	
091	DF6	Dokan	Sediment	A¯B¯CDT¯	ND	ND
	DF7	Dokan	Sediment	A¯B¯CDT¯	ND	ND
	DF10	Dokan	Sediment	A¯B¯CDT¯	ND	ND
	DF11	Dokan	Sediment	A¯B¯CDT¯	ND	ND
	DF4	Dokan	Sediment	A¯B¯CDT¯	ND	ND
	CD105KSE1	Dokan	Sediment	A¯B¯CDT¯	107	1
	CD105KSE2	Dokan	Sediment	A¯B¯CDT¯	107	
	CD105KSO10	Chnarok	Soil	A¯B¯CDT¯	107	
001	F1	Dokan	Sediment	A + B + CDT¯	ND	ND
	F5	Dokan	Sediment	A + B + CDT¯	ND	ND
	F7	Dokan	Sediment	A + B + CDT¯	ND	ND
	CD105KSE3	Dokan	Sediment	A + B + CDT¯	3	1
	CD105KSE4	Dokan	Sediment	A + B + CDT¯	3	
010	CD105KSE9	Hamamok	Sediment	A¯B¯CDT¯	15	1
	7M	Hamamok	Sediment	A¯B¯CDT¯	ND	ND
	2M	Hamamok	Sediment	A¯B¯CDT¯	ND	ND
	12M	Hamamok	Sediment	A¯B¯CDT¯	ND	ND
	6M	Hamamok	Sediment	A¯B¯CDT¯	ND	ND
011	F9	Dokan	Sediment	A + B + CDT¯	ND	ND
	CD105KSE6	Jalee	Sediment	A¯B¯ CDT¯	136	1
035	CD105KSE5	Jalee	Sediment	A¯B¯CDT¯	107	1
	CD105KSE11	Jalee	Sediment	A¯B¯CDT¯	107	

The RT designation was ascertained using capillary ribotyping targeting the 16S-23S rRNA intergenic spacer. Toxin profiles were determined using PCR to amplify the partial and essential repeat regions of toxin A genes, toxin B, and binary toxin genes.

### Diversity of the isolates based on ribotypes and toxin genes carriage

Six RTs: RT001 (five isolates), RT010 (five isolates), RT011 (two isolates), RT035 (two isolates), RT091 (eight isolates), and RT604 (nine isolates) were identified. Although the study only examined a small number of isolates, diverse RTs both the sites and within specific samples were observed (Table 1, Supplementary Table S1, Fig. S1). Characterizing the isolates based on the presence or absence of *C. difficile* toxin genes showed that of the 31 isolates, 25 (81%) were negative for both *tcd*A and *tcd*B (A_¯_B_¯_) genes, while the remaining six (19%) isolates encode the toxigenic (A+ B+) genes. The RT011 isolates from Dokan (F9) and Jalee (CD105KSE6) had contrasting toxin profiles with the latter being toxin negative, while the F9 strain encodes both toxin genes *tcd*A and *tcd*B (Table 1). Furthermore, all the strains were binary toxin-negative (CDT_¯_; Table 1). PCR amplicons were sequenced and shown to match the genome data.

### Diverse MLST profiles exist among the strains

To gain a detailed understanding of the genome characteristics of the representative RT isolates, a total of eleven isolates representing RT091 (three isolates), RT001, RT035, RT604 (two isolates each), and one isolate each from RT010 and RT011 were sequenced. The assembled genomes ranged from 49 to 458 contigs and 2594–2823 open reading frames per genome (as of February 2023; Supplementary Table S3), and their completeness and contamination level are shown in Supplementary Table S4.

Six distinct ST (ST-3, ST-15, ST-107, ST-137, ST-177, and ST-181) and seven RT/ST associations were identified from the isolates (Table 2). Five of the RT/ST associations (RT011/ST-137, RT035/ST-107, RT091/ST-107, RT604/ST-177, and RT604/ST-181) are novel, and the latter three are linked to RTs previously uncharacterized by MLST (Griffiths et al. 2010, Stabler et al. 2012, Dingle et al. 2014, Gawlik et al. 2015). The other two of the seven associations, RT001/ST-3 and RT010/ST-15, identified here were previously described among UK isolates (Stabler et al. 2012, Janezic and Rupnik 2015). Multiple STs, such as ST-177 and ST-181 were uniquely associated with RT604 (Table 2). In contrast, two RTs, RT091 and RT035, were associated with a single ST, ST-107, as shown in Table 2.

**Table 2.** Characteristic of allelic profiles (STs) of the 11 strains of *C. difficile* isolated in this study, and ST/RT associations.

MLST alleles										
Isolates	RT	ST	Clade	*adk*	*atpA*	*dxr*	*glyA*	*recA*	*sodA*	*tpi*
CD105KSE1	091	107	1	4	1	6	1	3	1	1
CD105KSE2	091	107	1	4	1	6	1	3	1	1
CD105KSE3	001	3	1	1	1	2	1	1	1	1
CD105KSE4	001	3	1	1	1	2	1	1	1	1
CD105KSE5	035	107	1	4	1	6	1	3	1	1
CD105KSE6	011	137	1	1	1	2	3	1	3	1
CD105KS07	604	177	C-I	13	18	22	33	18	31	28
CD105KS08	604	181	C-I	13	18	22	31	18	31	26
CD105KSE9	010	15	1	1	1	6	1	8	5	1
CD105KSO10	091	107	1	1	4	1	6	1	3	1
CD105KSE11	035	107	1	1	4	1	6	1	3	1

Five novel RT/ST associations: 011/137, 035/107, 091/107, 604/ST177, and 604/181 are reported in this study.

### Phylogenetic relationships based on core genome

**Figure 1.**
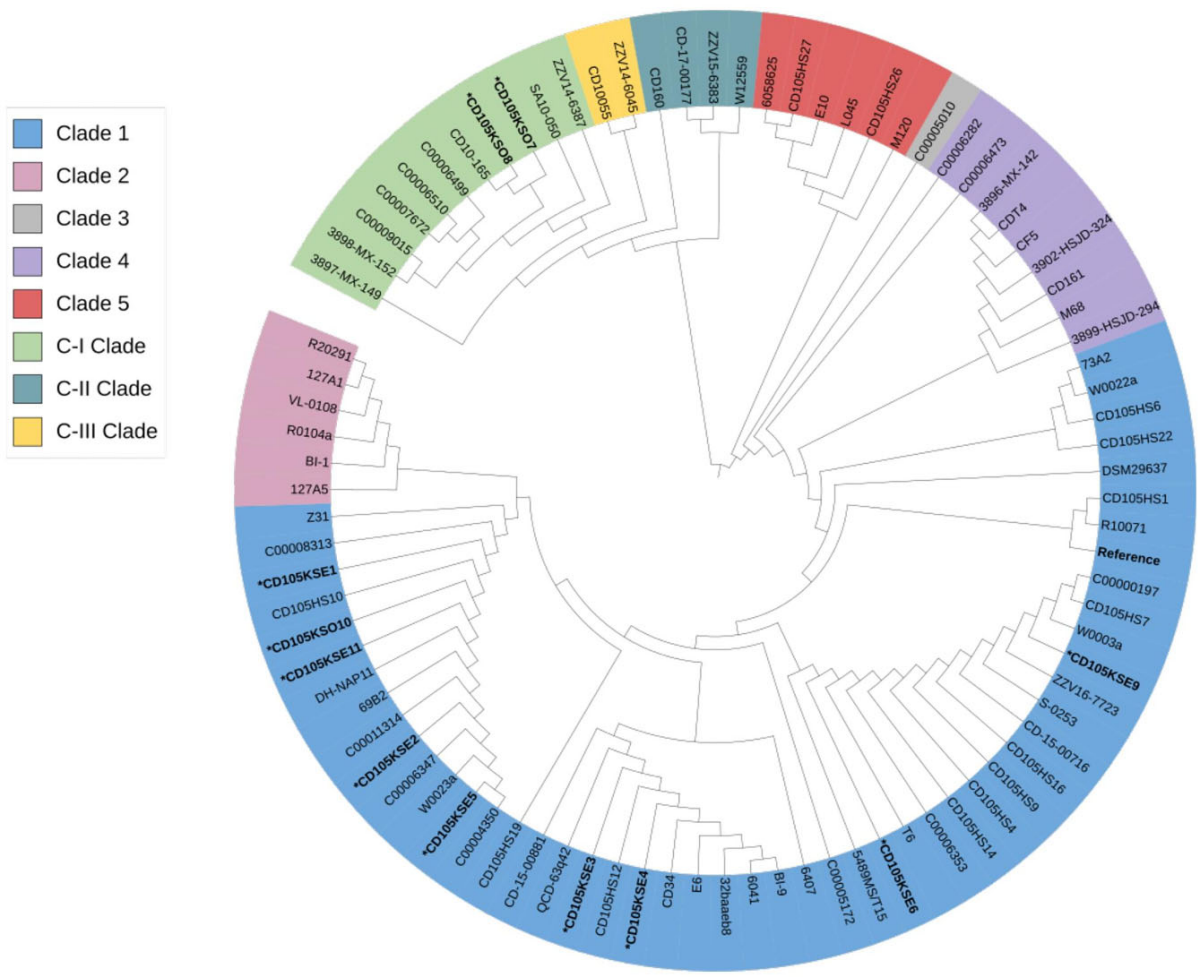
Phylogenetic tree showing five clades (1, 2, 3, 4, and 5) and three cryptic clades (C-I, C-II, and C-III) of *C. difficile* isolates examined based on core genome comparison. Maximum likelihood tree was constructed based on the core genes of 11 strains examined in this study (*) and 78 other reference *C. difficile* using PhyML as described previously. Single nucleotide polymorphisms (SNPs) in core genes were utilized for the phylogeny, and recombination was accounted for with clonalframeML. Tree visualized in an iTOL software v6.4.3.

We explored the phylogenetic relationships and diversity of our eleven sequenced strains in the context of other 78 publicly available *C. difficile* genomes from diverse geographical regions comprising of 28 RTs and 29 strain types of the known eight clades (1, 2, 3, 4, 5, C-I, C-II, and C-III; Supplementary Table S2). Phylogenetic analysis based on whole-genome alignment revealed eight discrete (Clades 1, 2, 3, 4, and 5) and the three previously observed deeply branching clades (Clades C-I, C-II, and C-III; Squire et al. 2015, Ramírez-Vargas et al. 2018, Knight et al. 2021; Fig. 1).

Consistent with other findings, Clade 1 is the most diverse comprising of seventeen RTs, sixteen STs, and includes toxigenic and non-toxigenic isolates (Supplementary Table S2; Janezic and Rupnik 2015, Janezic et al. 2016). Nine of the isolates characterized in this study (CD105KSE1, CD105KSE2, CD105KSE3, CD105KSE4, CD105KSE5, CD105KSE6, CD105KSE9, CD105KSO10, and CD105KSE11) belonged to Clade 1. *Clostridioides difficile* strains CD105KSE3, CD105KSE4, CD105KSE5, and CD105KSE11 closely cluster with the other strains of the same RT. However, CD105KSE6 (RT035) clusters distantly from strains of the similar RT.

The Clade C-I, reclassified as a novel independent *Clostridioides* genomospecies with C-II and C-II clades comprised of RT206, RT289, RT290, RT127, and RT604 isolates, in addition to six other isolates. The RT604 isolates from this study (CD105KSO7 and CD105KSO8) are new additions to the C-I Clade and the only strains from environmental source with the rest being of clinical origin within this clade (Fig. 1).

### Multiple prophage carriage detected in environmental strains of *C. difficile*

We explored the genomes of the isolates and multiple intact and partial prophages were detected within the genomes of the strains (Supplementary Table S5). The size of the intact prophages ranged from 20.6 to 137.9 kb, while the incomplete prophages ranged from 6.8 to 62.1 kb (SupplementaryTable S5). Two intact prophages were identified in CD105KSE1, CD105KSE2, and CD105KSO10, while three intact prophages were found in CD105KSE3, CD105KSE4, CD105KSE5, CD105KSO7, and CD105KSE11. Strains CD105KSE9, CD105KSO8, and CD105KSSE6 had five, four, and one intact prophages predicted in their genomes, respectively. Further analysis of all the predicted regions of the intact prophages in the isolates using BLAST showed similarity to other *C. difficile* phages (Supplementary Table S5).

### CRISPR-Cas system diversity in environmental *C. difficile* strains

**Figure 2.**
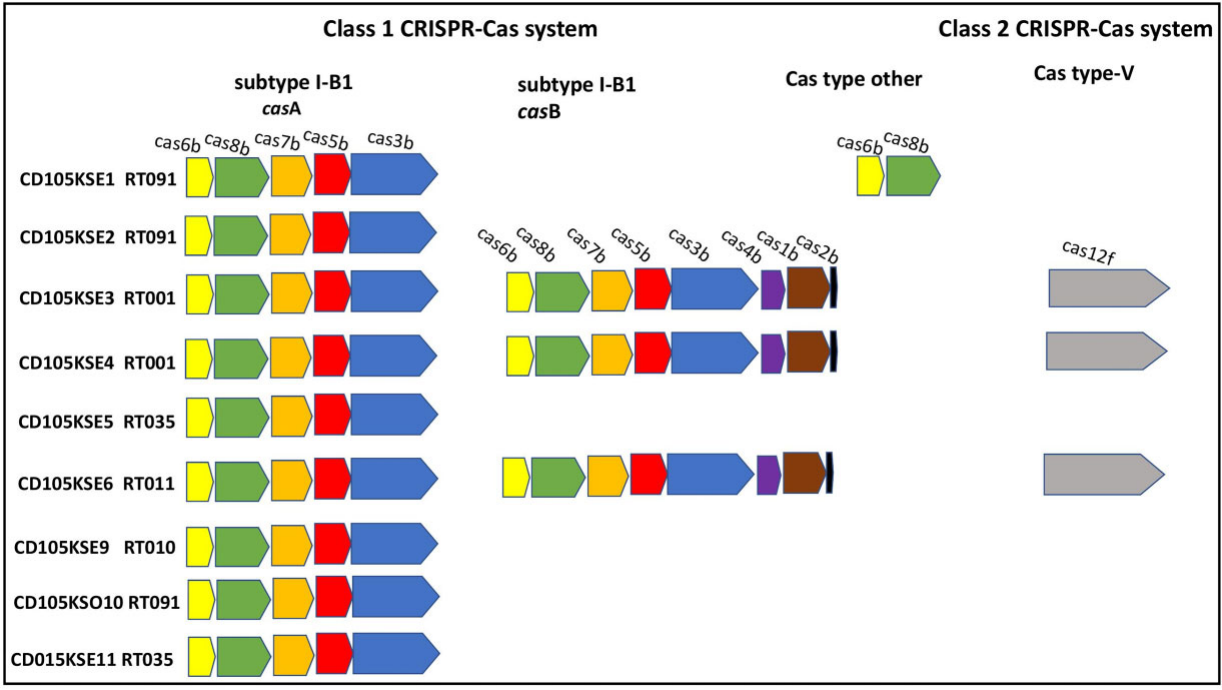
Schematic diagram showing the CRISPR-Cas systems carried by environmental *C. difficile* isolates examined in this study. Typical operon organization is shown for each CRISPR-Cas system. Class 1 subtype I-B1 Cas system identified in 81.8% of the genomes code for the two mainly conserved clusters of *cas* genes that identified in this type. Cluster *cas*A that encodes partial *cas* gene set (*cas*b*6, casb8, casb*7, *casb*5, and *casb*3), and cluster *cas*B codes for a complete set of subtype I-B1 *cas* genes (*cas*b*6, casb8, casb*7, *casb*5, and *casb*3) and (*casb*1, *casb*2, and *casb*4). Class 2 V-type Cas system founded in 27.3% of the genomes encode for a single large effector protein (*cas*12f). Cas type other founded in only 9% of the genomes has only two genes homologous to *cas6b* and *cas8b*. Homologous *cas* genes are shown with coloured arrows. Colour coding is the same for homologous *cas* genes.

The genomes of the eleven strains were also screened for the presence of CRISPR-Cas systems and found to encode multiple CRISPR arrays, ranging from three to twelve per genome (Fig. 2), with a variable number of DR (average length ∼29 bp) separated by variable spacer contents, ranging from 45 to 112 per strain. A total of 97 DRs were extracted from the CRISPR arrays of the 11 strains, and 22 different DR consensus sequences were identified. Of these, six consensus DRs were unique (Supplementary Fig. S2). SNPs and identical DR sequences are also observed within the arrays of multiple strains (Supplementary Fig. S2). The observed variation is possibly expected assuming how widespread the system is (Rath et al. 2015).

CRISPR-Cas systems were defined within the genomes of *C. difficile* strains, two known classes of CRISPR-Cas systems (class 1 subtype I-B1 and class 2 type V CRISPR-Cas systems), and a CRISPR-Cas type that has two genes homologous to *cas6b* and *cas8b* only were identified (Fig. 2; Payne et al. 2021). Class 1 subtype I-B1 CRISPR-Cas system is described by multi-subunit protein effectors and was previously observed in all queried genomes of *C. difficile* strains (Hargreaves et al. 2014, Boudry et al. 2015, Andersen et al. 2016, Maikova et al. 2018, Maikova et al. 2019). Class 2 type V CRISPR-Cas systems possess single, large protein effectors (Makarova et al. 2015), observed in several bacterial genomes (Schunder et al. 2013, Vestergaard et al. 2014), and also known as genome editing system that comprises of crRNA and *Cas*12a protein (Liu et al. 2020). No *cas* genes were identified in the genome of RT604 strains CD105KSO7 and CD105KSO8. This lack of *cas* genes could be due to the deletion through horizontal gene transfer resulting in several independent deletions of the complete set of *cas* genes as shown in enterococcal strains (Palmer and Gilmore 2010).

In the subtype I-B1 CRISPR-Cas system, the two mainly conserved clusters of *cas* genes were identified, consistent with an earlier report (Andersen et al. 2016). The first *cas* gene cluster, termed *cas*A, encodes a partial *cas* gene set (*cas*b*6, casb8, casb*7, *casb*5, and *casb*3) lacking *casb*1, *casb*2, and *casb*4 and was identified in 81.8% of the genomes. The second *cas* gene cluster is *cas*B, which encodes a complete set of subtype I-B1 *cas* genes (*cas*b*6, casb8, casb*7, *casb*5, and *casb*3) as well as (*casb*4, *casb*1, and *casb*2) identified in 27.3% of sequenced strains (Andersen et al. 2016, Maikova et al. 2018; Fig. 2). *Cas* operons incidence was found to be associated with the RT profiles; for example, strains of RT091, RT035 and RT010 have similar cas gene clusters, *cas*A, that encode a partial *cas* gene set (Fig. 2). Class 2 V-type Cas system with a single large effector protein with 1380 amino acid lengths (Cas12f) has been found in 27.3% of the studied genomes (Pyzocha and Chen 2018, Xiao et al. 2020). Diversity was observed within the strains based on the multiple CRISPR-Cas types; 36.4% of the strains encode two different types of CRISPR-Cas systems within a single genome. For example, both strains of RT001 and strain of RT010 have class 1 subtype I-B1 and class 2 type V CRISPR-Cas systems. Interestingly, the RT091 strain, CD105KSE1, encodes subtype-I-B1 with a *casA* gene cluster and another CRISPR-Cas type with an unknown Cas-type that has only two *cas* genes encoded for *casb*6 and *casb*8 (Fig. 2).

### CRISPR spacers homology among the *C. difficile* strains

**Figure 3.**
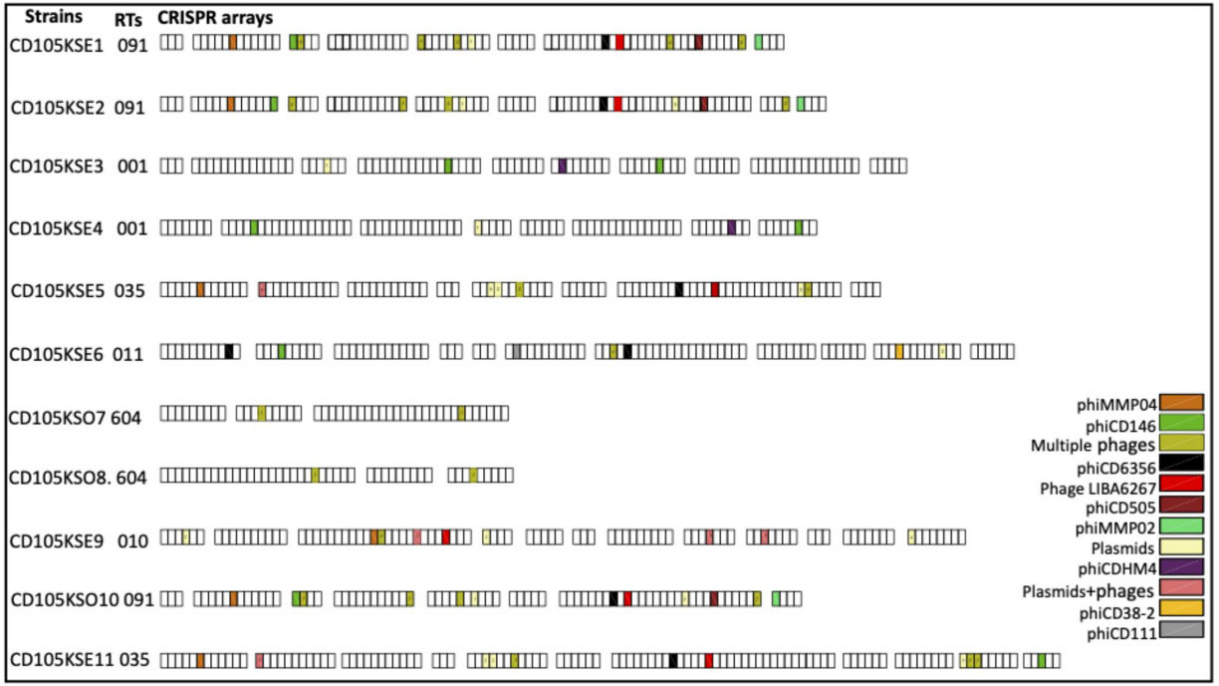
CRISPR arrays and the corresponding identical spacers encoded in *C. difficile* isolates examined in this study. Identical sequences between spacers and phage sequences are indicated by matching colours; each colour represents the similarity with a particular *C. difficile* phage (see legend). Coloured spacers with numbers correspond to multiple of protospacers that match *C. difficile* phages and plasmids sequences as shown in Supplementary Table S6. (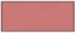 refers to the spacers that match to plasmid and *C. difficile* phage sequences, the numbers refer to the number of matched plasmid and *C. difficile* phage sequences, 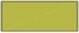 refers to the spacers that match to *C. difficile* phage sequences, the numbers refer to the number of matched phages, and 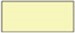 refers to spacers that target multiple plasmid sequences, the numbers refer to the number of matched plasmids). White boxes represent spacers that have no homology to any *C. difficile* phages.

To determine if the CRISPR-Cas systems of the 11 characterized strains could target known phages, the spacers of the arrays within the genomes of the strains were searched against Genbank and BLAST nucleotide databases and RefSeq-Plasmid databases using the CRISPRTarget tool (Biswas et al. 2013). In total, 1054 spacers were identified from the genome of our strains, of which 185 were identical to other published *C. difficile* phages and plasmid sequences from a diverse range of geographical locations, and 869 spacers were novel (Supplementary Table S6). From the 185 identical spacers, 118 spacers were identical to other published *C. difficile* phages, 67 spacers identical to plasmid sequences (as of March 2023; Fig. 3, Supplementary Table S6). Strains of RT001, RT091, and RT604 share a similar spacer sequence identity consistent with their evolutionary relationships (Boudry et al. 2015). Both RT604 isolates have the lowest number of spacers (45 spacers), and both strains have three CRISPR arrays with the same number of spacers in each array. However, only two spacers from the arrays of both strains have sequences similar to other published *C. difficile* phages. This suggests that the majority of the spacers might be derived from unknown phages that have yet to be isolated or characterized. Spacer numbers 13 and 39 in CD105KSO7 have sequences similar to spacer numbers 40 and 22 of strain CD105KSO8, respectively. We have observed conserved numbers of arrays and spacers among the three strains of RT091, but strain CD105KSE2 has only one extra array with four spacers (Fig. 3, Supplementary Table S6). Whilst RT035 strains shared some common spacers, CD105KSE5 lacked the spacer for phiCD146, suggesting that dynamic changes in the CRISPR array content had occurred, possibly through interactions with foreign DNA elements (Hargreaves et al. 2014). It was observed that more than one CRISPR spacer within a strain from all RTs targeted the same phage. For example, two spacers (31, 73) from different CRISPR arrays from strain CD105KSE1 targeted phiCD27, signifying constant interactions between this strain and the corresponding phage (Boudry et al. 2015). Some of the strains carry multiple spacers for the same phage, such as CD105KSE3, which has spacer 12 and spacer 79 showing identical matches to phiCD146. Spacers for phiCDHM19 were only observed in two strains, CD105KSE5 and CD105KSE11, suggesting a less widespread predicted immunity of this strain to this phage (Mayer et al. 2008, Horgan et al. 2010, Meessen-Pinard et al. 2012, Sekulovic et al. 2014; Fig. 3). The shared spacers could also imply probable hot spots of phage genome evolution loci in which bacterial strains are more exposed to these phages, which have counter-evolved through infections.

## Discussion

The paucity of information on environmental *C. difficile* strains in the Middle East compared to strains from western countries reflects the lack of study in this area. Typically, this pathogen has been considered to be a problem in the western world, and thus, it has not been a priority in other places. However, new geographical areas such as Slovenia and Thailand are beginning to explore this pathogen from different environmental sources to understand the diversity that exists amongst the strains (Janezic et al. 2016, Putsathit et al. 2017, Imwattana et al. 2020, Tkalec et al. 2020).

The work conducted in Slovenia reported *C. difficile* prevalence from puddle water (14.4%, *n *= 104), soil (36.7%, *n *= 79), meat (3.6%, *n *= 336), and raw vegetables (6.1%, *n *= 98). Majority of the isolates identified represent RTs (such as RT002, RT005, RT010, RT014/020, RT015, and RT023), which may suggest their association with humans and animals (Janezic et al. 2016, Tkalec et al. 2020). The studies from Thailand focussed on clinical settings and reported prevalence of 9.2% and 15.6% (*n* = 422) of toxigenic and nontoxigenic isolates, respectively, from the samples (Imwattana et al. 2020, Tkalec et al. 2020).

The very limited studies on this pathogen in the Middle East have focused on characterizing isolates from clinical/hospitals sources (Khalil et al. 2019, Shoaei et al. 2019, Al-Tawfiq et al. 2020, Azimirad et al. 2020, Baghani et al. 2020, Williamson et al. 2022), raw meat (Esfandiari et al. 2014, Bakri 2018, Ersoz Seyma and Cosansu 2018), food products (Rahimi et al. 2015, Bakri 2016), and supermarket environments (Sadeghifard et al. 2010, Shoaei et al. 2019). In the Middle East region, the reported prevalence rates of CDI are 23.8% in Jordan, 8%–10% in Kuwait, and 5.15% in Saudi Arabia (Alzouby et al. 2020). However, there are no surveillance strategies to show the occurrence of CDI in northern Iraq. The lack of information on the strains that are found in the region’s natural environment, or potentially transmitted by human and animal activities may greatly affect the control of this infection in this region and the world at large. Also, environmental *C. difficile* strains have been reported to encode several genetic elements that could contribute to the emergence of novel clinical strains in hospitals, as previously reported (Hargreaves et al. 2015).

We previously reported the genome characteristic of three novel species of *Clostridia* from the natural habitats of this region, in which all three isolates encode multiple prophage elements and the CRISPR Cas-system was found in two of the isolates (Rashid et al. 2016). Here, we went further to isolate and characterize *C. difficile* isolates from river sediments and soils in northern Iraq for further work in this area.

In the current study, we isolated *C. difficile* from the sediment and soil samples of four of the seven examined sites in northern Iraq. This indicates that these sources are important habitats from which to study *C. difficile* presence and diversity, which concurs with previous work conducted in our laboratory and elsewhere (al Saif and Brazier 1996, Hargreaves et al. 2013, Janezic et al. 2016, Rodriguez et al. 2019, Williamson et al. 2022). Again, consistent with previous work, here, the highest number of isolates that yielded *C. difficile* (21/31 isolates, ∼68%) were from sediment samples. This may be attributed to the dormant spores, which protect the bacteria and therefore may contribute to the transmission and persistence of *C. difficile* in the marine ecosystem (Zidaric et al. 2010, Hargreaves et al. 2013, Xu et al. 2014).

Despite the small sample size, we had good recovery rates of *C. difficile*, which we isolated from four of the seven examined sites (∼60%), suggesting it is abundant in the types of areas. This rate of *C. difficile* isolation is comparable to a previous study, in which were 54% and 60% recovery were observed in two consecutive years (Hargreaves et al. 2013). However, one other study was unable to detect *C. difficile* from sediment samples (Pasquale et al. 2011), and others detected only 24.0% from environmental samples (Janezic et al. 2016). The bacterial recovery observed in our study may also be attributed to enrichment procedures carried out on the samples before isolation, which greatly enhanced the isolation of the bacterium (Hargreaves et al. 2013).

Both environmentally associated RTs (RT010, RT035, RT091, and RT604) isolates and those associated with an important clinical RT (RT001, RT011) were detected in our sample sites, which concurs with previous studies (Hargreaves et al. 2013, Hargreaves et al. 2016, Janezic et al. 2016). Whilst RT001, RT010, and RT035 have previously been isolated from the environments of Europe, all studies conducted in the Middle East have associated these RTs with clinical samples (Al-Tawfiq and Abed 2010, Hargreaves et al. 2013, Al-Thani et al. 2014, Azimirad et al. 2020, Baghani et al. 2020). To our knowledge, this is the first time that these RTs have been found to be associated with the environmental sources in these parts of the country. This may be linked to human activities such as recreational activities in all the sample areas and agricultural runoff found in Dokan. In contrast, none of the environmental RT strains reported by other researchers from the Middle East were isolated in this study. This may be attributed to the fact that some of the previously reported strains were not ribotyped, hence their identities are unknown (Jamal et al. 2002, Rotimi et al. 2003, Rahimi et al. 2015). In addition, pathogenic toxin strains that are associated with community-acquired infections were isolated from retail surfaces, which enhanced the need to understand their medical impact and to enact any necessary preventative measures (Alqumber 2014). We found uncommon RTs, RT604 and RT091 from the West to be prevalent in the examined areas at the time of our sampling. In contrast, RT010, which is common in both Europe and the United Kingdom, was found to be rare in the region examined (Rotimi et al. 2003, Al-Thani et al. 2014, Baghani et al. 2020). This suggests that certain *C. difficile* strains are more prevalent in certain regions of the world than others are.

The isolation of both toxigenic and nontoxigenic isolates in this study is consistent with previous studies (Rotimi et al. 2003, Hargreaves et al. 2013, Janezic et al. 2016). The diverse toxin gene profiles observed within a RT show that the pathogenicity locus is variable and may not be a feature of clonality, and could be readily lost (Dingle et al. 2014). We did not isolate any binary toxin-positive environmental isolates. However, RT078 and RT027 isolates, which encode for a binary toxin, have previously been identified in environmental samples from England and Saudi Arabia and may be attributed to human or animal activities (Hargreaves et al. 2013, Bakri 2016).

The isolation of six RTs that are associated with six MLST profiles also concurs with previous studies that showed that although MLST are normally associated with a specific RT, but may not always predict the strain types and *vice versa* (Griffiths et al. 2010, Wang et al. 2018, Zhao et al. 2021). Multiple RTs RT091 and RT0235 are associated with ST-107, and multiple STs ST-181 and ST-177 are related to RT604. The associations of RTs with multiple STs or *vice versa* have previously been reported and may suggest the constant divergent nature of *C. difficile* genomes (Dingle et al. 2011, Stabler et al. 2012, Janezic and Rupnik 2015, Janezic et al. 2016, Knight et al. 2017). Phylogenetic analysis of these strains isolated in this study identified a lineage (Clade C-I) that is highly divergent from the other five established clades. In line with earlier studies, Clade 1 is diverse in term of RTs and STs, and comprised of both toxigenic and nontoxigenic strains (Stabler et al. 2012, Janezic and Rupnik 2015, Janezic et al. 2016, Ramírez-Vargas et al. 2018).

The occurrence of multiple and diverse prophage carriage within *C. difficile* is high and has been previously isolated from environmental strains (Shan et al. 2012, Hargreaves et al. 2013, Hargreaves et al. 2015, Mullany et al. 2015). Here, we detected up to six intact prophages in a single *C. difficile* genome, and this complex network of prophages within environmental strains could contribute to the evolution of new pathogenic strains. Further work is required to ascertain if all the six prophages are inducible, as observed in previous work (Fortier and Sekulovic 2013, Hargreaves et al. 2015).

Evidence of the interplay between hosts and phages can be seen from the CRISPR arrays detected here. The CRISPR-Cas system is a form of adaptive immunity that bacteria use to resist phage infection (Hargreaves et al. 2014, Maikova et al. 2018). Our results show that this system is diverse within our strains (Soutourina et al. 2013, Hargreaves et al. 2014, Hargreaves et al. 2016). Here, we showed for the first time the prevalence of the class 2 type V CRISPR-Cas system in *C. difficile* strains. To date, class 1 subtype I-B is the native CRISPR-Cas system in *C. difficile* (Boudry et al. 2015, Maikova et al. 2018, Maikova et al. 2019). Both strains of RT001 possess a class 2 type V CRISPR-Cas system present within the mobile genetic region in both strains, this perhaps had been acquired via horizontal gene transfer as studies proposed that class 2 effectors originated from nuclease encoded by different mobile genetic elements (MGE; Koonin and Makarova 2019). We have also reported the presence of two or more CRISPR-Cas types within the genome of a single strain; 27.3% of the sequenced strains carry class 2 type V CRISPR-Cas systems beside the native subtype I-B CRISPR-Cas systems (Fig. 2). Multiple CRISPR-Cas systems have been found in some organisms that occur naturally (Carte et al. 2014). Consistent with the earlier reports, both *cas* gene sets (*casA* and *casB*) of the I-B subtype were found within the sequenced strain (Boudry et al. 2015, Maikova et al. 2018). The occurrence of *cas* operons found to be associated with the RT profiles. The variation of the CRISPR-Cas system types and the contents within RT strains could affect their susceptibility to infection by phages (Hargreaves et al. 2014). The spacer contents of CRISPR arrays are identical to known phage sequences and are particularly insightful since it was previously shown that 100% identity between spacer and proto-spacer sequences is required to provide immunity (Boudry et al. 2015, Maikova et al. 2018, Deem 2020). Although small numbers of mismatches could confer a degree of immunity during infection through target cleavage (Michael et al. 2022). Our data are in line with earlier studies and supports the potential role of phages to drive the evolution of epidemic strains (Hargreaves and Clokie 2014).

## Conclusions

To conclude, *C. difficile* strains were found to be present in the natural environment of northern Iraq and were readily isolated from 57% samples obtained. Genome analysis showed that these strains are diverse and distinct from those found elsewhere, and as is seen in all *C. difficile* genomes, these strains had multiple prophage carriages with diverse CRISPR-Cas system types that have arrays containing diverse spacers. We have showed for the first time instance of the class 2 type V CRISPR-Cas system in *C. difficile* strains that has been described in other bacterial genomes. Although this was a small-scale study, the observations of RT and genome diversity in this region would provide an overall understanding of the diversity of this organism. Studies in new geographies will further reveal insights into how this pathogen can evolve and increase our understanding on the relationship between strains observed in patients and those found in the environment.

## Supplementary Material

fnad091_Supplemental_Files
