# Supplementary material for: Novel ribotype/sequence type associations and diverse CRISPR-Cas systems in environmental Clostridioides difficile strains from northern Iraq
Source: FEMS Microbiol Lett. 2023 Sep 18;370:fnad091. doi: 10.1093/femsle/fnad091 (PMC10806358; doi:10.1093/femsle/fnad091)
Supplement: fnad091_Supplemental_Files [file fnad091_supplemental_files.zip › Supplemental files_.docx]

Table S1. Details of sampling sites and samples. A refer to sediment sample, B refer to soil sample.

| Dates of collection | Sites | No of Sample | | GPS Coordinates | | Distance from the nearest residential area |
| --- | --- | --- | --- | --- | --- | --- |
|  |  | **Soil** | **Sediment** | **Latitude** | **Longitude** |  |
| 14/02/2013 | Hamamok | 1 | 1 | 36.09695 | 44.608709 | 4-6km |
| 1/08/2012 | Dokan | B | A | 35.949559 | 44.962103 | 4-7 km |
| 3/11/2013 | Jalee river | 1 | 1 | 36.189427, | 44.609946 | 4-7km |
| 3/11/2012 | Chnarok | 1 | 0 | 36.11416 | 44.65868 | 111-12 km |
| 3/11/2013 | Taq taq river | 1 | 1 | 35.85510 | 44.55268 | 8.5-9km |
| 1/08/2012 | Safeen mountain | A, B | 0 | 36.40455 | 44.29705 | 11.8-12km |
| 3/11/2013 | Haibat Sultan Mountain | 1 | 0 | 36.101834 | 44.670839 | 9-10 km |

**Table S2. *C. difficile* genomes used to establish phylogenetic relationship with the novel strains (*) described in this study.**

| **ST** | **Uberstrains** | **RT** | **Strain ID** | **Accession** | **Clade** | **Isolation Source** | **Place** |
| --- | --- | --- | --- | --- | --- | --- | --- |
| ND | CLO_AA4851AA | ND | 127A1 | PRJNA344770 |  | Environment | USA |
| ND | CLO_AA4849AA | ND | 127A5 | PRJNA344770 |  | Environment | USA |
| ND | CLO_AA0534AA | 078 | CD105HS27 | PRJEB8943 |  | Environment | UK |
| ND | CLO_AA0530AA | 078 | CD105HS26 | PRJEB8943 |  | Environment | UK |
| ND |  | ND | E10 | CAME00000000 (**NCBI**) |  | Horse | Slovenia |
| 37 | CLO_AA9063AA | ND | CD161 | NZ_CP029154.1 | 4 | Human | China |
| ND |  | ND | CD160 | SAMN01766591 |  | Human | USA |
| ND | CLO_AA8032AA | ND | ZZV16-7723 | PRJNA430032 |  | Shoe sole | Slovenia |
| ND | CLO_BA9985AA | 010 | CD-15-00716 | PRJEB33779 |  | Human | Germany |
| ND | CLO_AA0535AA | 010 | CD105HS9 | PRJEB8943 |  | Environment | UK |
| ND | CLO_AA0529AA | 010 | CD105HS16 | PRJEB8943 |  | Environment | UK |
| ND | CLO_AA0537AA | 031 | CD105HS19 | PRJEB8943 |  | Environment | UK |
| ND | CLO_AA0541AA | 012 | CD105HS1 | PRJEB8943 | 1 | Environment | UK |
| ND | CLO-BA0214AA | ND | CD105HS14 | PRJEB8943 |  | Environment | UK |
| ND | CLO_AA6040AA | ND | E6 | PRJEB3957 |  | Human | Canada |
| 200 |  | ND | W12559 | SAMN05219384 (**NCBI**) | C-II | Human | UK |
| ND | CLO_AA0536AA | 001 | CD105HS12 | PRJEB8943 |  | Environment | UK |
| ND | CLO_BA9937AA | 001 | CD-15-00881 | PRJEB33779 |  | Human | Germany |
| ND | CLO_AA0539AA | 005 | CD105HS10 | PRJEB6886 |  | Environment | UK |
| ND | CLO_AA0533AA | 002 | CD105HS7 | PRJEB8943 |  | Environment | UK |
| ND | CLO_AA4854AA | ND | 69B2 | PRJNA344770 |  | Human | USA |
| 13 |  | 095 | T6 | CAMR00000000(**NCBI**) | 1 | Human | Hungary |
| ND | CLO_AA0540AA | 014 | CD105HS4 | PRJEB6886 |  | Environment | UK |
| ND | CLO_AA4852AA | ND | 73A2 | DAEPKT000000000 |  | Human | USA |
| ND | CLO_AA0532AA | 220 | CD105HS22 | PRJEB8943 |  | Environment | UK |
| ND | CLO_AA0531AA | 220 | CD105HS6 | PRJEB8943 |  | Environment | UK |
| 1 | CLO_AA5207AA | 027 | BI-1 | PRJEB2039 | 2 | Human | UK |
| 1 | CLO_AA9077AA | ND | R0104a | PRJNA224116 | 2 | Human | USA |
| 1 | CLO_AA4162AA | 027 | R20291 | PRJNA40921 | 2 | Human | UK |
| 2 | CLO_AA9075AA | ND | W0022a | PRJNA224116 | 1 | Human | USA |
| 3 | CLO-AA4172AA | 001 | BI-9 | FN668944 | 1 | Human | USA |
| 83 | CLO-AA6446AA | 032 | DSM29637 | SAMN05188782 | 1 | Human | Indonesia |
| 3 | CLO_AA0703AA | 001 | *CD105KSE3 | PRJEB8702 | 1 | Environment | Iraq |
| 3 | CLO_DA1476AA | 001 | *CD105KSE4 |  | 1 | Environment | Iraq |
| 3 | CLO_AA6481AA | 009 | Z31 | PRJNA224116 | 1 | Dog | Brazil |
| 5 | CLO_AA3436AA | 069 | C00005010 | PRJEB3010 | 3 | Human | UK |
| 8 | CLO_AA9074AA | ND | W0003a | PRJNA224116 | 1 | Human | USA |
| 11 | CLO_AA3441AA | 045 | L045(C00002490) | SAMEA1487096 | 5 | Human | Library isolate |
| 11 | CLO-AA6485AA | 078 | M120 | FN665653 | 5 | Human | UK |
| 13 | CLO_AA5222AA | 020 | R10071 | PRJNA248340 | 1 | Environment | Poland |
| 15 |  | ND | 5489MS/T15 | NZ_MOPF00000000(**NCBI**) | 1 | Human | USA |
| 15 | CLO_DA1478AA | 010 | *CD105KSE9 |  | 1 | Environment | Iraq |
| 15 | CLO_AA3435AA | 010 | C00005172 | PRJEB3010 |  | Human | UK |
| 23 | CLO_AA3431AA | 138 | C00006282 | ERP001417 | 4 | Human | UK |
| 36 | CLO_AA3437AA | 011 | C00004350 | PRJEB3010 | 1 | Human | UK |
| 86 | CLO_AA6486AA | 017 | CF5 | PRJNA158359 | 4 | Human | Belgium |
| 37 | CLO_AA4163AA | 017 | M68 | FN668375 | 4 | Human | Ireland |
| 40 | CLO_AA4834AA | 035 | C00006347 | SAMEA2052296 | 1 | Human | UK |
| 42 |  | 106 | DH/NAP11/106/ST-42 | PRJNA393704 | 1 | Human | USA |
| 42 | CLO_AA9076AA | ND | W0023a | NZ_CP025045.1 | 1 | Human | USA |
| 54 | CLO_AA6490AA | 012 | **Reference** (CD630) | PRJNA57679 | 1 | Human | Switzerland |
| 77 | CLO_AA3460AA | 011 | C00000197 | PRJEB3010 | 1 | Human | UK |
| 107 | CLO_AA0696AA | 035 | *CD105KSE11 | PRJEB8702 | 1 | Environment | Iraq |
| 107 | CLO_AA0702AA | 035 | *CD105KSE5 | PRJEB8702 | 1 | Environment | Iraq |
| 107 | CLO_AA0706AA | 091 | *CD105KSE1 | PRJEB8702 | 1 | Environment | Iraq |
| 107 | CLO_AA0705AA | 091 | *CD105KSE2 | PRJEB8702 | 1 | Environment | Iraq |
| 107 | CLO_AA0697AA | 091 | *CD105KSO10 | PRJEB8702 | 1 | Environment | Iraq |
| 107 | CLO_AA4830AA | ND | C00011314 | PRJEB1483 | 1 | Human | UK |
| 137 | CLO_AA0701AA | 011 | *CD105KSE6 | PRJEB8702 | 1 | Environment | Iraq |
| 11 | CLO-AA5219AA | 126 | 6058625 | SAMN02904068 | 5 | Environment | Poland |
| ND | CLO_AA9511AA | 001 | 32baaeb8 | PRJNA398458 |  | Human | Hungary |
| 170 | CLO_AA4822AA | ND | C00006473 | SAMEA2052309 | 4 | Human | Australia |
| ND |  | ND | 6407 | ADEH00000000(**NCBI**) |  | Equine | USA |
| ND | CLO-AA6340AA | ND | 6041 | AVIP01000001.1 |  | Equine | USA |
| 177 | CLO_DA1477AA | 604 | *CD105KSO7 |  | C-I | Environment | Iraq |
| 177 | CLO_AA4821AA | ND | C00007672 | SAMEA2052310 | C-I | Human | UK |
| 179 | CLO_AA4819AA | 289 | C00006510 | SAMEA2052311 | C-I | Human | Australia |
| 180 | CLO_AA4818AA | 290 | C00006499 | SAMEA2052312 | C-I | Human | Australia |
| 181 | CLO_AA0699AA | 604 | *CD105KSO8 | PRJEB8702 | C-I | Environment | Iraq |
| 181 | CLO_AA4817AA | 127 | C00009015 | SAMEA2052313 | C-I | Human | Australia |
| 181 | CLO_AA4982AA | ND | CD10-165 | JRHN01000001 | C-I | Human | France |
| 8 |  | 002 | S-0253 | CP076401(**NCBI**) |  | Human | Australia |
| 206 | CLO_AA4983AA | ND | SA10-050 | PRJNA260039 | C-I | Human | France |
| 359 |  | ND | 3897-HMX149 | https://microbesng.uk /portal/projects/149D9096-03DF-4C6B-BF5F-2119D1AE7B68 | C-I | Human | Costa Rica |
| ND |  | ND | 3896-HMX-142 |  |  | Human | Costa Rica |
| 360 |  | ND | 3898-HMX-152 |  | C-I | Human | Costa Rica |
| ND |  | ND | 3899-HSJD-294 |  |  | Human | Costa Rica |
| ND |  | ND | 3902-HSJD-324 |  |  | Human | Costa Rica |
| ND | CLO-AA1096AA | ND | CD34 | FUTM00000000 |  | Environment | UK |
| 343 | CLO_AA8746AA | ND | ZZV14-6045 | ERR2216003 | C-III | Human | Germany |
| 338 | CLO_CA5848AA | ND | ZZV15-6383 | JAGWCV000000000.1 | C-II | Environment | Slovenia |
| ND | CLO_CA1593AA | ND | VL_0108 | FAAL00000000.1 |  | Human | Canada |
| ND | CLO_CA5852AA | ND | ZZV14-6387 | SAMN17865203 |  | Environment | Slovenia |
| 369 |  | ND | CD10055 | ERR2215981 (**NCBI**) | C-III | NA | France |
| ND | CLO_AA2069AA | ND | C00008313 | ERR340252 |  | Human | UK |
| 637 | CLO_AA7299-AA | 126 | CD-17-00177 | ERR3296451 | C-II | Human | Spain |
| 3 |  | ND | QCD-63q42 | ABHD00000000.2(**NCBI**) | 1 | Human | Canada |
| 37 | CLO-AA9062AA | ND | CDT4 | NZ_CP029152.1(**NCBI**) | 4 | Animal | China |
| 48 | CLO-AA3429A | 38 | C00006353 | SAMEA1487061 | 1 | Human | UK |

**Figure S1. Diversity and distribution of isolates and ribotypes within the *C. difficile*- positive sites in Northern Iraq. A,** Number of ribotype isolates identified. **B**, Dendrogram of *C. difficile* isolates based on the size and copy number of the 16S-23S rRNA inter spacer region amplicons as determined by capillary based ribotyping, ribotype/sequence type association of the strains. The similarity of the strains was assessed using a MultiVariate statistical package (MVSP) clustering analysis based on the presence or absence of bands of a particular size.

**Table S3. Summary of *C. difficile* genomes**

**Table S4. Summary of CheckM analysis on *C. difficile* genomes.**

| **Strains** | **Marker Lineage** | **Genomes** | **Markers** | **Marker Sets** | **0** | **1** | **2** | **3** | **4** | **5+** | **Completeness** | **Contamination** | **N50** |
| --- | --- | --- | --- | --- | --- | --- | --- | --- | --- | --- | --- | --- | --- |
| CD105KSE1 | Clostridiales | 304 | 250 | 143 | 0 | 245 | 5 | 0 | 0 | 0 | 100 | 2.47 | 60588 |
| CD105KSE2 | Clostridiales | 304 | 250 | 143 | 0 | 249 | 1 | 0 | 0 | 0 | 100 | 0.23 | 180336 |
| CD105KSE3 | Clostridiales | 304 | 250 | 143 | 0 | 250 | 0 | 0 | 0 | 0 | 100 | 0 | 161100 |
| CD105KSE4 | Clostridiales | 304 | 250 | 143 | 0 | 250 | 0 | 0 | 0 | 0 | 100 | 0 | 20512 |
| CD105KSE5 | Clostridiales | 304 | 250 | 143 | 0 | 250 | 0 | 0 | 0 | 0 | 100 | 0 | 277022 |
| CD105KSE6 | Clostridiales | 304 | 250 | 143 | 0 | 250 | 0 | 0 | 0 | 0 | 100 | 0 | 190962 |
| CD105KSO7 | Clostridiales | 304 | 250 | 143 | 0 | 250 | 0 | 0 | 0 | 0 | 100 | 0 | 324718 |
| CD105KSO8 | Clostridiales | 304 | 250 | 143 | 0 | 250 | 0 | 0 | 0 | 0 | 100 | 0 | 88365 |
| CD105KSE9 | Clostridiales | 304 | 250 | 143 | 0 | 246 | 4 | 0 | 0 | 0 | 100 | 2.1 | 442314 |
| CD105KSO10 | Clostridiales | 304 | 250 | 143 | 0 | 250 | 0 | 0 | 0 | 0 | 100 | 0 | 79714 |
| CD105KSE11 | Clostridiales | 304 | 250 | 143 | 0 | 249 | 1 | 0 | 0 | 0 | 100 | 0.17 | 147778 |

Table S5. Prophage predictions in the genomes of the sequenced *C. difficile* strains.

| **Strains** | **Ribotypes** | **Score** | **Prophage Region** | **Length (Kb)** | **Completeness** | **Region position** | **Possible phage** |
| --- | --- | --- | --- | --- | --- | --- | --- |
| **CD105KSE1** | **091** | 50 | 1 | 46.6 | Incomplete | 34516-81151 | PHAGE_Faecal_FP_Toutatis_NC_047915(2) |
|  |  | 150 | 2 | 43.9 | Intact | 28662-72576 | PHAGE_Clostr_phiCDHM19_NC_028996(32) |
|  |  | 40 | 3 | 18.1 | Incomplete | 16610-34728 | PHAGE_Escher_vB_ESCOS_NC_047776 (4) |
|  |  | 40 | 4 | 27.3 | Incomplete | 13048-40377 | PHAGE_Clostr_phiCDHM19_NC_028996(10) |
|  |  | 40 | 5 | 22.4 | Incomplete | 7394-29830 | PHAGE_Clostr_phiCT453A_NC_028991(7) |
|  |  | 100 | 6 | 29 | Intact | 636-29677 | PHAGE_Clostr _CDMH1_NC_024144 (24) |
|  |  | 70 | 7 | 58.1 | questionable | 90-58266 | PHAGE_Clostr_CDKM15_NC_048643(31) |
| **CD105KSE2** | **091** | 50 | 1 | 46.6 | Incomplete | 35331-81966 | PHAGE_Faecal_FP_Toutatis_NC_047915(2) |
|  |  | 150 | 2 | 53.2 | Intact | 52250-105460 | PHAGE_ Clostr_phiCDHM19_NC_028996(35) |
|  |  | 40 | 3 | 18.1 | Incomplete | 122776-140894 | PHAGE_ Escher _vB-EcoM-Schickermooser_ _NC_048196 (4) |
|  |  | 40 | 4 | 27.3 | Incomplete | 45018-72347 | PHAGE_Clostr_ phiCDHM19_NC_028996(10) |
|  |  | 120 | 5 | 50.8 | Intact | 12335-63176 | PHAGE_Clostr_phiC2_NC_00923(26) |
|  |  | 70 | 6 | 58.1 | Questionable | 90-58266 | PHAGE_Clostr_CDKM15_NC_048643(31) |
| **CD105KSE3** | **001** | 20 | 1 | 10.9 | Incomplete | 58664-69595 | PHAGE_ Clostr_CDMH1_NC_024144(3) |
|  |  | 130 | 2 | 34.3 | Intact | 8945-43264 | PHAGE_ Clostr_phiCDHM19_NC_028996(12) |
|  |  | 40 | 3 | 20.7 | Incomplete | 6525-27275 | PHAGE_Clostr_phiCDHM19_NC_028996(9) |
|  |  | 30 | 4 | 13.7 | Incomplete | 158704-172416 | PHAGE_Clostr_CDKM9_NC_048642(4) |
|  |  | 130 | 5 | 137.9 | Intact | 3-137961 | PHAGE_Clostr_phiCD211_NC_029048(119) |
|  |  | 150 | 6 | 34 | Intact | 283-34308 | PHAGE_Clostr_CDMH1_NC_024144(19) |
|  |  | 40 | 7 | 9.6 | Incomplete | 3-9643 | PHAGE_Clostr_CDMH1_NC_024144(6) |
|  |  | 30 | 8 | 11.7 | Incomplete | 3-11704 | PHAGE_Clostr_CDMH1_NC_024144(9) |
| **CD105KSE4** | **001** | 30 | 1 | 13.7 | Incomplete | 483267-496979 | PHAGE_Clostr_CDKM9_NC_048642(4) |
|  |  | 10 | 2 | 23.6 | Incomplete | 11696-35299 | PHAGE_Clostr_phiCT19406A_NC_030950(3) |
|  |  | 40 | 3 | 20.6 | Incomplete | 139891-160548 | PHAGE_Clostr_phiCDHM19_NC_028996(9) |
|  |  | 140 | 4 | 33.6 | Intact | 134044-167655 | PHAGE_Clostr_CDMH1_NC_024144(18) |
|  |  | 177 | 5 | 137.9 | Intact | 1-137911 | PHAGE_Clostr_phiCD211_NC_029048(119) |
|  |  | 120 | 6 | 34.3 | Intact | 976-35295 | PHAGE_Clostr_phiCDHM19_NC_028996(12) |
|  |  | 30 | 7 | 11.6 | Incomplete | 919-12572 | PHAGE_Clostr_CDMH1_NC_024144(9) |
|  |  | 40 | 8 | 9.5 | Incomplete | 2214-11806 | PHAGE_Clostr_CDMH1_NC_024144(6) |
| **CD105KSE5** | **035** | 40 | 1 | 27.3 | Incomplete | 44798-72114 | PHAGE_ Clostr_phiCDHM19_NC_028996(10) |
|  |  | 150 | 2 | 45.4 | Intact | 12265-57746 | PHAGE_Clostr_phiMMP01_NC_028883(11) |
|  |  | 50 | 3 | 25 | Incomplete | 210-25288 | PHAGE_Clostr_phiCD505_NC_028764(10) |
|  |  | 50 | 4 | 62 | Incomplete | 96518-158603 | PHAGE_Clostr_phiCDHM14_NC_048665(24) |
|  |  | 30 | 5 | 13.7 | Incomplete | 210-13918 | PHAGE_Clostr_ CDMH1_NC_024144(7) |
|  |  | 100 | 6 | 22.4 | Intact | 618-23053 | PHAGE_Clostr_phiMMP01_NC_028883(21) |
|  |  | 110 | 7 | 21.9 | Intact | 616-22610 | PHAGE_Clostr_phiCDHM19_NC_028996(26) |
| **CD105KSE6** | **011** | 50 | 1 | 23 | Incomplete | 3-23057 | PHAGE_Clostr_CDMH1_NC_024144(11) |
|  |  | 40 | 2 | 27.3 | Incomplete | 152774-180080 | PHAGE_Clostr_phiCDHM19_NC_028996(11) |
|  |  | 30 | 3 | 13.7 | Incomplete | 299695-313408 | PHAGE_Clostr_CDKM9_NC_048642(4) |
|  |  | 30 | 4 | 17.6 | Incomplete | 487538-505145 | PHAGE_Clostr_phiCD506_NC_028838(2) |
|  |  | 20 | 5 | 39.2 | Incomplete | 1154-40380 | PHAGE_Clostr_ phiCDHM14_NC_048665(22) |
|  |  | 30 | 6 | 13.5 | Incomplete | 1387-14920 | PHAGE_Clostr_phiMMP03_NC_028959(10) |
|  |  | 20 | 7 | 13.4 | Incomplete | 3-13419 | PHAGE_Clostr_phiCT453A_NC_028991(7) |
|  |  | 90 | 8 | 16.2 | Questionable | 1-16231 | PHAGE_Clostr_phiCDHM19_NC_028996 (12) |
|  |  | 140 | 9 | 55.4 | Intact | 61415-116842 | PHAGE_Clostr_phiCDHM19_NC_028996 (16) |
|  |  | 30 | 10 | 28.7 | Incomplete | 146434-175209 | PHAGE_Clostr_phiCD6356_NC_015262(6) |
| **CD105SO7** | **604** | 10 | 1 | 16 | Incomplete | 233604-249664 | PHAGE_Clostr_CDKM9_NC_048642(3) |
|  |  | 70 | 2 | 31 | questionable | 235319-266382 | PHAGE_Clostr_phiMMPO2_NC_019421(8) |
|  |  | 60 | 3 | 27.9 | Incomplete | 311315-339263 | PHAGE_Clostr_phiCDHM19_NC_028996(9) |
|  |  | 60 | 4 | 9.7 | Incomplete | 1-9702 | PHAGE_Bacill_0305phi8_36_NC_009760(5) |
|  |  | 130 | 5 | 48.1 | Intact | 67652-115762 | PHAGE_Clostr_phiMMP01_NC_028883(12) |
|  |  | 60 | 6 | 25.2 | Incomplete | 952-26224 | PHAGE_Clostr_phiCD211_NC_029048(4) |
|  |  | 50 | 7 | 26.8 | Incomplete | 119856-146699 | PHAGE_Clostr_phiCDHM19_NC_028996(7) |
|  |  | 60 | 8 | 21.1 | Incomplete | 46-21232 | PHAGE_Clostr_CDKM9_NC_048642(15) |
|  |  | 40 | 9 | 13.9 | Incomplete | 6-13987 | PHAGE_Clostr_CDMH1_NC_024144(10) |
|  |  | 90 | 10 | 31.3 | Questionable | 3-31378 | PHAGE_Clostr_phiCD506_NC_028838 (8) |
|  |  | 100 | 11 | 29.8 | Intact | 3-29851 | PHAGE_Clostr_phiMMP03_NC_028959(25) |
|  |  | 50 | 12 | 24.6 | Incomplete | 1-24688 | PHAGE_Clostr_phiCD6356_NC_015262(6) |
|  |  | 10 | 13 | 23.4 | Incomplete | 793-24244 | PHAGE_Clostr_phiCD27_NC_011398(16) |
|  |  | 120 | 14 | 24.1 | Intact | 424-24541 | PHAGE_Clostr_phiCDHM19_NC_028996(26) |
| **CD105KSO8** | **604** | 110 | 1 | 30.2 | Intact | 2-30297 | PHAGE_Clostr_phiMMP03_NC_028959(25) |
|  |  | 60 | 2 | 25.2 | Incomplete | 1125-26397 | PHAGE_Clostr_phiCD211_NC_029048(4) |
|  |  | 10 | 3 | 24.2 | Incomplete | 128-24372 | PHAGE_Clostr_CDKM9_NC_048642(17) |
|  |  | 110 | 4 | 23.6 | Intact | 664-24320 | PHAGE_Clostr_phiCDHM19_NC_028996(25) |
|  |  | 40 | 5 | 28 | Incomplete | 268160-296201 | PHAGE_Clostr_phiCDHM19_NC_028996(10) |
|  |  | 60 | 6 | 16.8 | Incomplete | 340869-357695 | PHAGE_Clostr_CDKM15_NC_048643(9) |
|  |  | 10 | 7 | 22.3 | Incomplete | 358722-381029 | PHAGE_Clostr_CDKM9_NC_048642(3) |
|  |  | 60 | 8 | 28.5 | Incomplete | 119905-148492 | PHAGE_Clostr_phiCDHM19_NC_028996(7) |
|  |  | 150 | 9 | 45.4 | Intact | 794-46196 | PHAGE_Clostr_phiCDHM19_NC_028996(16) |
|  |  | 150 | 10 | 58.7 | Intact | 19347-78130 | PHAGE_Clostr_phiCDHM19_NC_028996(16) |
|  |  | 40 | 11 | 6.8 | Incomplete | 76601-83489 | PHAGE_Clostr_phiCD6356_NC_015262(2) |
|  |  | 60 | 12 | 10.3 | Incomplete | 52742-63135 | PHAGE_Clostr_CDKM9_NC_048642(15) |
|  |  | 60 | 13 | 9.7 | Incomplete | 1-9750 | PHAGE_Bacill_0305phi8_36_NC_009760(5) |
|  |  | 130 | 14 | 48.2 | Intact | 67700-115900 | PHAGE_Clostr_phiMMP01_NC_028883(12) |
|  |  | 40 | 15 | 26.2 | Incomplete | 33964-60215 | PHAGE_Clostr_CDMH1_NC_024144(10) |
|  |  | 40 | 16 | 11.4 | Incomplete | 1-11416 | PHAGE_Bacil_SP_NC_031245(1) |
| **CD105KSE9** | **091** | 30 | 1 | 13.3 | Incomplete | 245386-258733 | PHAGE_Clostr_CDKM9_NC_048642(4) |
|  |  | 60 | 2 | 27.4 | Incomplete | 320925-348368 | PHAGE_Clostr_phiCDHM19_NC_028996(11) |
|  |  | 40 | 3 | 18.1 | Incomplete | 68553-86671 | PHAGE_ Escher_ESCO13-047770(4) |
|  |  | 150 | 4 | 35.3 | Intact | 103802-139179 | PHAGE_Clostr_phiMMP02_NC_019421(32) |
|  |  | 130 | 5 | 33.9 | Intact | 73466-107376 | PHAGE_Clostr_phiCD211_NC_029048 (6) |
|  |  | 150 | 6 | 46.9 | Intact | 2-46943 | PHAGE_Clostr_phiMMP01_NC_028883(37) |
|  |  | 116 | 7 | 26.3 | Intact | 1054-27420 | PHAGE_Clostr_phiCD506_NC_028838 (41) |
|  |  | 110 | 8 | 25.8 | Intact | 3-25882 | PHAGE_Clostr_phiCDHM19_NC_028996 (32) |
| **CD105KS010** | **091** | 50 | 1 | 46.6 | Incomplete | 35413-82048 | PHAGE-Faecal_FP_Toutatis_NC_047915 (2) |
|  |  | 150 | 2 | 50 | Intact | 36085-86157 | PHAGE_Clostr_phiCDHM19_NC_028996 (35) |
|  |  | 40 | 3 | 18.1 | Incomplete | 21380-39498 | PHAGE_Escher_vB_EcoM_Schickermooser_NC_048196(4) |
|  |  | 40 | 4 | 27.3 | Incomplete | 44872-72201 | PHAGE_Clostr_phiCDHM19_NC_028996(10) |
|  |  | 120 | 5 | 50.8 | Intact | 9928-60769 | PHAGE_Clostr_CDMH1_NC_024144(26) |
|  |  | 70 | 6 | 58.1 | questionable | 90-58266 | PHAGE_Clostr_CDKM15_NC_048643(31) |
| **CD105KS11** | **035** | 50 | 1 | 46.6 | Incomplete | 35856-82491 | PHAGE_Faecal_FP_Toutatis_NC_047915(2) |
|  |  | 40 | 2 | 27.3 | Incomplete | 24874-52190 | PHAGE_Clostr_phiCDHM19_NC_028996(10) |
|  |  | 150 | 3 | 45.6 | Intact | 18932-64563 | PHAGE_Clostr_phiMMP01_NC_028883(15) |
|  |  | 50 | 4 | 25 | Incomplete | 210-25288 | PHAGE_Clostr_phiCD505_NC_028764(12) |
|  |  | 50 | 5 | 62.1 | Incomplete | 96599-158698 | PHAGE_Clostr_phiCDHM14_NC_048665(24) |
|  |  | 30 | 6 | 13.7 | Incomplete | 210-13918 | PHAGE_Clostr_CDMH1_NC_024144(7) |
|  |  | 100 | 7 | 22.4 | Intact | 658-23084 | PHAGE_Clostr_phiMMP01_NC_028883(21) |
|  |  | 100 | 8 | 20.6 | Intact | 1075-21748 | PHAGE_Clostr_phiCDHM19_NC_028996(24) |

**Strains Consensus DR sequences**

**
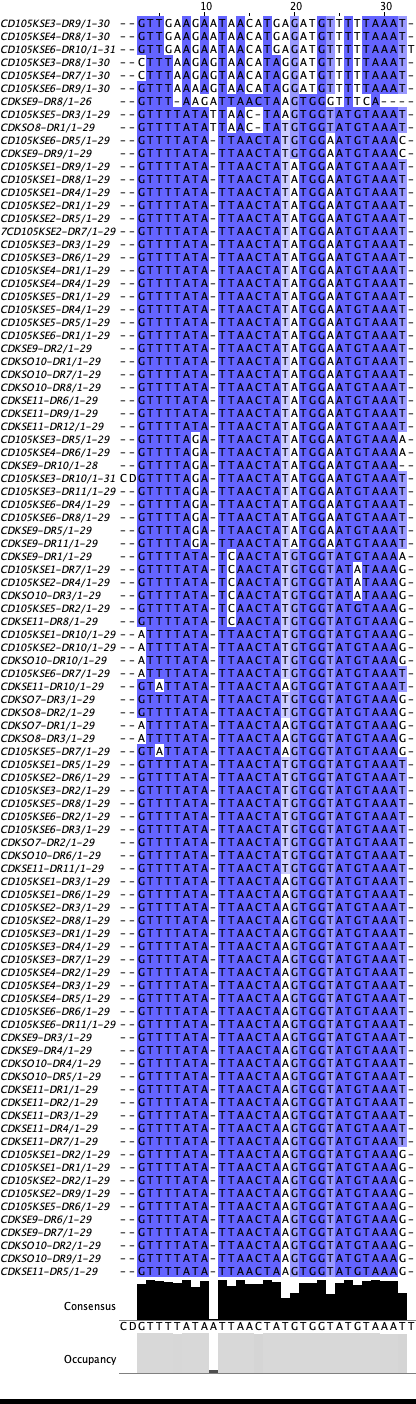
**

**Figure S2.** **Alignment of the consensus DR from each CRISPR arrays.** Arrays are colour coded reflect conserved groups. Single polynucleotide polymorphisms (SNPs); white boxes.
